# The Need for Person-Centered Measures for Dementia Research and Care

**DOI:** 10.1093/geroni/igab046.1008

**Published:** 2021-12-17

**Authors:** Sam Fazio, Sheryl Zimmerman, Laura Gitlin

## Abstract

The importance of person-centered medical and psychosocial care has become widely recognized, but there is abundant evidence that care is not always person-centered. In 2018, the Alzheimer’s Association published their evidence-informed Dementia Care Practice Recommendations, which address nine domains all grounded in a person-centered perspective. Following that work, the Association launched LINC-AD -- Leveraging an Interdisciplinary Consortium to Improve Care and Outcomes for Persons Living with Alzheimer's and Dementia. An early effort of LINC-AD, and the focus of this symposium, examined what measures are available to guide care and assess outcomes, and the extent to which they embrace person-centeredness. The results have been disappointing. This session will highlight the importance of person-centered measures in five domains of the Dementia Care Practice Recommendations, based on comprehensive reviews of literature. Each paper, presented by LINC-AD research advisors, will examine available measures and raise questions about gaps using a person-centered lens. Katie Maslow will describe frequently used measures and identify person-centered measures that could be added to studies of alternate procedures intended to increase detection and diagnosis. Drs. Mast and Molony will discuss a person-centered approach to item development and testing for assessment. Emilee Ertle will discuss the need to measure interpersonal and contextual factors associated with behavioral expressions. Drs. Prizer and Zimmerman will compare measures of dressing ability and their person-centered components. Dr. Calkins will examine the strengths and limitations of environmental assessment tools. As Discussant, Dr. Gitlin will integrate the findings from all five presentations, suggesting directions for the future.

